# Prenatal maternal stress and birth outcomes in rural Ghana: sex-specific associations

**DOI:** 10.1186/s12884-019-2535-9

**Published:** 2019-10-29

**Authors:** Kenneth Ayuurebobi Ae-Ngibise, Blair J. Wylie, Ellen Boamah-Kaali, Darby W. Jack, Felix Boakye Oppong, Steven N. Chillrud, Stephaney Gyaase, Seyram Kaali, Oscar Agyei, Patrick L. Kinney, Mohammed Mujtaba, Rosalind J. Wright, Kwaku Poku Asante, Alison G. Lee

**Affiliations:** 1 Ghana Health Service, Kintampo Health Research Centre, Brong Ahafo Region Kintampo, Ghana; 2 School of Medicine and Public Health, University of Newcastle, Callaghan, New South Wales Australia; 3 Division of Maternal-Fetal Medicine, Department of Obstetrics and Gynecology, Beth Israel Deaconess Medical Center, Boston, MA USA; 4 Department of Environmental Health Sciences, Mailman School of Public Health, Columbia University, New York, NY 10032 USA; 5 Lamont-Doherty Earth Observatory at Columbia University, Palisades, NY USA; 6 Department of Environmental Health, Boston University School of Public Health, Boston, MA USA; 7 Department of Environmental Medicine and Public Health, Icahn School of Medicine at Mount Sinai, New York, NY USA; 8 Department of Pediatrics, Kravis Children’s Hospital, Icahn School of Medicine at Mount Sinai, New York, NY USA; 9 Division of Pulmonary, Critical Care and Sleep Medicine, Icahn School of Medicine at Mount Sinai, 1468 Madison Avenue, New York, NY 10029 USA

**Keywords:** Negative life events, Prenatal maternal stress, Birth anthropometrics, Birth outcomes, Sex-specific effects

## Abstract

**Background:**

In developed countries, prenatal maternal stress has been associated with poor fetal growth, however this has not been evaluated in rural sub-Saharan Africa. We evaluated the effect of prenatal maternal stress on fetal growth and birth outcomes in rural Ghana.

**Methods:**

Leveraging a prospective, rural Ghanaian birth cohort, we ascertained prenatal maternal negative life events, categorized scores as 0-2 (low stress; referent), 3-5 (moderate), and > 5 (high) among 353 pregnant women in the Kintampo North Municipality and Kintampo South District located within the middle belt of Ghana. We employed linear regression to determine associations between prenatal maternal stress and infant birth weight, head circumference, and length. We additionally examined associations between prenatal maternal stress and adverse birth outcome, including low birth weight, small for gestational age, or stillbirth. Effect modification by infant sex was examined.

**Results:**

In all children, high prenatal maternal stress was associated with reduced birth length (*β* = − 0.91, *p* = 0.04; *p*-value for trend = 0.04). Among girls, moderate and high prenatal maternal stress was associated with reduced birth weight (*β* = − 0.16, *p* = 0.02; *β* = − 0.18, p = 0.04 respectively; *p*-value for trend = 0.04) and head circumference (*β* = − 0.66, *p* = 0.05; *β* = − 1.02, *p* = 0.01 respectively; *p*-value for trend = 0.01). In girls, high prenatal stress increased odds of any adverse birth outcome (OR 2.41, 95% CI 1.01-5.75; p for interaction = 0.04). Sex-specific analyses did not demonstrate significant effects in boys.

**Conclusions:**

All infants, but especially girls, were vulnerable to effects of prenatal maternal stress on birth outcomes. Understanding risk factors for impaired fetal growth may help develop preventative public health strategies.

**Trial registration:**

NCT01335490 (prospective registration).

**Date of Registration:** April 14, 2011.

**Status of Registration:** Completed.

## Background

Low birth weight, a global marker of intrauterine growth, is an independent predictor of early-neonatal, late-neonatal and post-neonatal infant mortality, even after accounting for gestational age at delivery [1]. Head circumference and birth length also reflect intrauterine growth. Approximately 98% of the world’s perinatal mortality occurs in low- and middle-income countries with the highest rates in sub-Saharan Africa [2–4]. The importance of fetal growth extends beyond perinatal mortality-- the Developmental Origins of Health and Disease (DOHaD) theory posits that fetal growth predicts disease risk across the life course, including cardiovascular risk and cognitive decline [5–7]. Therefore, understanding risk factors contributing to reductions in birth weight, head circumference, and/or length may help identify at-risk pregnancies and targets for public health interventions.

In higher-income countries, maternal psychosocial stress during or prior to pregnancy has been associated with low birth weight, prematurity, intrauterine growth retardation and other negative birth outcomes [8–10]. To our knowledge, only one prospective study has examined the associations between prenatal maternal stress, with a focus on childhood trauma and lifetime post-traumatic stress disorder, and infant birth outcomes in a peri-urban sub-Saharan Africa and found that increased maternal trauma adversely impacted fetal growth [11]. However, maternal stress during pregnancy in rural sub-Saharan Africa has not previously been described. While mechanisms are not well delineated, stress-associated disruption in key regulatory systems in the pregnant woman, including hypothalamic-pituitary-adrenal (HPA) axis, autonomic nervous system (ANS), and immune functioning may lead to altered fetal development and enhanced vulnerability to low birth weight.

Sex-specific effects of maternal prenatal stress on birth outcomes have also been reported. Prior work suggests that female fetuses differentially adapt to intrauterine stressors by decreasing growth rates. A study in Israel of chronic maternal stress found that female fetuses were at increased risk for preterm birth and low birth weight [12]. Similarly, in Chile, maternal prenatal exposure to a negative life event was associated with a reduction in gestational age and increased risk of preterm birth, especially in girls [13]. Females may have increased HPA axis reactivity and female placentas may increase permeability to glucocorticoids following maternal stress, as compared to males [14]. Sex-specific differences in placental gene expression and immune function have also been described [15, 16].

We therefore leveraged a prospective pregnancy cohort to examine the impact of prenatal maternal stress as indexed by negative life events (NLEs) on infant birth anthropometrics and birth outcomes in rural Ghana. We hypothesized that infants born to women with higher levels of prenatal stress would have impaired birth anthropometrics and increased risk of adverse birth outcomes.

## Methods

### Study participants

The study was conducted in the Kintampo North Municipality and Kintampo South District located within the middle belt of Ghana. Subjects were recruited from the Ghana Randomized Air Pollution and Health Study (GRAPHS), which has been described elsewhere [17]. Briefly, between August 2013 and March 2016, GRAPHS enrolled 1414 non-smoking, pregnant women from Kintampo North Municipality and Kintampo South District in rural central Ghana. Gestational age at enrollment was confirmed by ultrasound and all households were enrolled prior to 24 weeks gestation [18]. Following enrollment, women were randomized to one of three cooking strategies and followed for the remainder of their pregnancy and the infant’s first year of life.

The analysis in this manuscript include 353 pregnant women enrolled in GRAPHS who were in the third trimester of pregnancy between July 2014 to June 2015, and after informed consent underwent an additional maternal stress assessment in the third trimester of pregnancy. All enrolled participants had birth anthropometrics available for analysis. Procedures were approved by human studies and institutional ethics committees at Kintampo Health Research Centre (KHRC), the Icahn School of Medicine at Mount Sinai and the Columbia University Mailman School of Public Health and written consent was obtained from all study participants.

### Negative life events

Stress theory postulates that when environmental demands exceed coping strategies, we experience stress and consequent physiological disruption. Vulnerability to stress and physiological disruption is increased when events are experienced across multiple domains. Prenatal maternal stress was measured in the third trimester of pregnancy using the Crisis in Family Systems-Revised (CRISYS-R) survey, suitable for use in lower-income settings [19]. The CRISYS-R tool was used in this study because it measures negative life events likely to be experienced by lower-income and mixed ethnic populations [19–21]. The CRISYS-R survey has been validated in English and Spanish [22, 23], and used in studies across populations including Africa [11]. Pregnant women were asked to endorse life events experienced in the prior 6 months across 11 domains, including financial, legal, relationships, career, safety in the community, safety in the home, medical issues pertaining to others, medical issues pertaining to self, authority, home issues, and prejudice. Pregnant women were asked to rate each endorsed event as positive, negative, or neutral. The numbers of domains with at least one negative event were summed to create a negative life events (NLEs) domain score, with higher scores suggesting increased stress.

### Birth anthropometrics and outcomes

All birth anthropometrics were measured within 24 h of birth. Birth weight was measured to the nearest gram using the Tanita BD 585 digital baby scale (Tokyo, Japan) after standardizing with a 1-kg (kg) weight. Birth length and head circumference were also measured to the nearest 0.1 cm (cm) using a standard measuring board and a Lasso-o™ respectively. Low birth weight (LBW) was defined as less than 2500 g. Gestational age at delivery was determined using ultrasound-established dates [18]. Small-for-gestational age (SGA) was defined as birth weight less than the 10th percentile [24]. A stillborn was considered if there were no signs of life at delivery.

### Covariates

Maternal age at enrollment, marital status, ethnicity and family home ownership were obtained through questionnaires at GRAPHS enrollment. Maternal height to the nearest 0.1 cm and weight to the nearest 0.1 kg were recorded at enrollment. Infant sex was recorded at delivery.

### Statistical analysis

As above, the CRYSIS-R assesses maternal stress across 11 domains; a negative life events (NLE) domain score is created by summing the number of domains with at least one reported negative event. The NLE score was categorized into three categories (low, moderate, high) around the study-determined interquartile range, or 0-2, 3-5, and > 5 respectively. First, we used univariate and multivariable linear regression models to examine associations between prenatal maternal stress and birth anthropometrics. Models were adjusted for covariates linked to stress and birth anthropometrics in prior research, including infant sex, gestational age at delivery, ethnicity, maternal age, weight, height, marital status, and socioeconomic status as measured by family home ownership. We additionally adjusted for GRAPHS cluster, as post-randomization differences in socioeconomic status between groups were noted, and study cluster was directly related to cookstove use (prenatal air pollution exposure). Effect modification by infant sex was examined in stratified analyses and by fitting interaction terms. Trend tests were performed.

We then employed univariate and multivariable logistic regression to determine associations between prenatal maternal stress and adverse birth outcomes, including LBW, SGA, and a composite variable of infants born with LBW or SGA or stillbirth. For these analyses, the moderate (NLE 3-5) and high (NLE > 5) prenatal maternal stress groups were collapsed together to maintain adequate cell sizes. Models were adjusted for infant sex, ethnicity, cluster, maternal age, weight, height and marital status and socioeconomic status. A sensitivity model additionally adjusted for gestational age at delivery. Effect modification by infant sex was again examined in stratified analyses and by fitting interaction terms.

Main effects were considered statistically significant if the *p*-value was less than 0.05. In subgroup analyses, interaction terms were considered suggestive of an interaction if the p-interaction was less than 0.10. Analyses were performed in R version 3.3.3 (Vienna, Austria).

## Results

Participant characteristics are summarized in Table 1. Prenatal maternal stress was prevalent with 177 (50%) and 79 (22%) pregnant women reporting moderate or high stress. Pregnant women most commonly reported stress in financial [*N* = 286 (81%)], relationship [*N* = 236 (67%)], and home issue [*N* = 247 (70%)] domains (Additional file 1: Table S1). Pregnant women were median age 28 years old; 56% were married and 54% reported home ownership. The two most common reported ethnicities were Dagarti (22%) and Akan (20%). Of the 353 infants, 188 (53%) were girls. Median gestational age at delivery was 39.9 weeks. Median birth weight was 3.00 kg, head circumference 33.8 cm, and birth length 47.5 cm. An adverse birth outcome (LBW, SGA, or stillbirth) was observed in 85 (24%) infants. Boys had larger head circumference and a trend to increased birth weight as compared to girls, otherwise there were no differences between sexes (Table 1).

**Table 1:** Participant characteristics

	All Infant (*N* = 353)		Girls (*N* = 188)		Boys (*N* = 165)	
Continuous variables, Median, IQR						
Maternal age (years)	28.3	23.4-34	28.7	23.5-33.9	27.1	23.3-34.3
Maternal height (cm)	155	152-160	155.1	151-162	155	152-160
Maternal weight (kg)	55	51-62	55	50-61	55.1	51-62
Gestational Age at delivery (weeks)	39.9	39.1-4.9	39.9	39.1-40.9	39.9	39.1-40.6
Infant birth weight (kg)	3.0	2.7-3.2	2.9	2.6-3.2	3.0	2.7-3.3
Infant head circumference (cm)	33.8	32.6-35	33.5	32.4-34.5	34.0	33-35
Infant birth length (cm)	47.5	45.1-49	47.1	45-49	48.0	45.6-49
Categorical variables, N, %						
Negative life events^a^						
0-2	97	27.5	56	51.6	41	24.8
3-5	177	50.1	87	46.3	90	54.5
> 5	79	22.3	45	23.9	34	20.6
Ethnicity						
Dagarti	79	22.4	47	25.0	32	19.4
Akan	71	20.1	40	21.3	31	18.8
Konkonba	54	15.3	31	16.5	23	13.9
Gonja	42	11.9	18	9.6	24	14.5
Mo	41	11.6	20	10.6	21	12.7
Other	66	18.7	32	17.0	34	20.7
Adverse Birth Outcome^b^						
Low birth weight	46	13	27	14.4	19	11.5
Small for gestational age	73	20.7	48	25.5	25	15.2
Stillbirth	7	2	2	1.1	5	3
Any adverse birth outcome	85	24.1	52	27.7	33	20
Cluster^c^						
Liquified petroleum gas stove	76	17.2	39	20.7	37	22.4
BioLite stove	116	32.9	57	30.3	59	35.8
Three stone fire stove (control)	161	46.6	92	48.9	69	41.8
Marriage status						
Married	197	55.8	106	56.4	91	55.2
Living together	126	35.7	69	36.7	57	34.5
Single	30	8.5	13	6.9	17	10.3
Home ownership						
Yes	191	54.1	97	51.6	94	57

Abbreviations: *IQR* interquartile range; *LPG* liquid petroleum gas; *NLEs* negative life events; *N* number

Adverse birth outcome: stillbirth, low birth weight or small for gestational age

^a^ Negative life events defined by the Crisis in Family Systems-Revised (CRISYS-R) survey^19^

^b^ Low birth weight defined as less than 2500 g; Small-for-gestational age defined as birth weight < 10th percentile; Stillbirth defined as no signs of life at delivery. Any adverse birth outcome, defined as any LBW, SGA, or stillborn infant

^c^ Cluster as defined by randomly assigned cookstove arm in the Ghana Randomized Air Pollution and Health Study

### Relationship between prenatal maternal stress and infant birth anthropometrics

Table 2 presents univariate and multivariable results of the association between prenatal maternal stress and infant birth anthropometrics including birth weight, head circumference and birth length, considered separately. In the overall sample, multivariable analyses did not demonstrate an association between prenatal maternal stress and birth weight or head circumference. High (NLEs > 5) vs. low (NLEs 0-2) prenatal maternal stress was associated with reductions in birth length (*β* = − 0.91, *p* = 0.04) with a significant trend test (p-for-trend = 0.04).

**Table 2:** Associations between prenatal negative life events (NLEs) and infant anthropometrics: Linear regression

NLEs	Univariate Model		Multivariable-Adjusted Model^a^	
	*β* (SE)	*p*-value	*β* (SE)	*p*-value
Outcome: Birth weight (kg)				
0-2	Reference	NA	Reference	NA
3-5	0.01 (0.05)	0.82	−0.07 (0.05)	0.24
> 5	0.04 (0.07)	0.49	−0.01 (0.06)	0.88
Outcome: Head Circumference (cm)				
0-2	Reference	NA	Reference	NA
3-5	0.33 (0.26)	0.21	0.04 (0.26)	0.89
> 5	0.16 (0.32)	0.62	−0.19 (0.30)	0.53
Outcome: Birth length (cm)				
0-2	Reference	NA	Reference	NA
3-5	0.12 (0.38)	0.76	−0.25 (0.39)	0.53
> 5	−0.54 (0.46)	0.23	**−0.91 (0.45)**	**0.04**

^a^ Multivariable models adjusted for infant sex, gestational age at delivery, ethnicity, cluster, maternal marriage status, age, weight and height; and family home ownership

Text in bold have *p*-values <0.05

Table 3 presents sex-stratified analyses for the association between prenatal maternal stress on birth anthropometrics including birth weight, head circumference and birth length, considered separately. Among girls, moderate (NLE 3-5) and high (NLE > 5) prenatal maternal stress were associated with reduced birth weight (NLE 3-5 *β* = − 0.16, *p* = 0.02; NLE > 5 *β* = − 0.18, *p* = 0.04) while no association was seen in boys (p-interaction = 0.009). Similarly, among girls, moderate and high prenatal maternal stress were associated with reduced head circumference (NLE 3-5 *β* = − 0.66, *p* = 0.05**;** NLE > 5 *β* = − 1.02, *p* = 0.01) while no association was seen in boys (p-interaction = 0.006). An association was seen between high maternal prenatal stress and birth length in girls (NLE > 5, *β* = − 1.28, *\* 0.04) but not boys (p-interaction 0.84). Among girls, the trend test suggested an exposure-response relationship for all outcomes (birth weight p-for-trend = 0.04; head circumference p-for-trend = 0.01; birth length p-for-trend = 0.04).

**Table 3:** Associations between prenatal negative life events (NLEs) and birth anthropometrics stratified by sex: Linear regression^a^

NLEs	Girls^b^		Boys^c^		p-interaction
	*β* (SE)	*p*-value	*β* (SE)	*p*-value	
Outcome: Birth weight (kg)					
0-2	Reference		Reference		
3-5	**−0.16 (0.07)**	**0.02**	0.02 (0.08)	0.81	0.009
> 5	**−0.18 (0.08)**	**0.04**	0.12 (0.10)	0.21	
Outcome: Head Circumference (cm)					
0-2	Reference		Reference		
3-5	−0.66 (0.34)	0.05	0.65 (0.39)	0.10	0.006
> 5	**−1.02 (0.40)**	**0.01**	0.66 (0.48)	0.17	
Outcome: Birth Length (cm)					
0-2	Reference		Reference		
3-5	−0.26 (0.53)	0.62	−0.57 (0.60)	0.35	0.84
> 5	**−1.28 (0.62)**	**0.04**	−0.98 (0.74)	0.18	

^a^ Multivariable models adjusted for gestational age at delivery, cluster, ethnicity, maternal marriage status, age, weight and height; and family home ownership

^b^ Girls: 0-2 (*n* = 56), 3-5 (*n* = 87), > 5 (*n* = 45)

^c^ Boys: 0-2 (*n* = 41), 3-5 (*n* = 90), > 5 (*n* = 34)

### Relationship between prenatal maternal stress and infant adverse birth outcomes

Table 4 presents multivariable associations between prenatal maternal stress and adverse birth outcomes, including LBW, SGA, and any adverse birth outcome (LBW, SGA or stillbirth) in the sample as a whole and stratified by sex. In the sample as a whole, no association was seen between prenatal maternal stress and adverse birth outcomes. When stratified by sex, effects were only evident in girls. Girls born to women with high (NLE > 2) prenatal stress had 4-fold increased odds of LBW (OR 4.44, 95% CI 1.11-17.76) and 2-fold increased odds of any adverse birth outcome (OR 2.41, 95% CI 1.01-5.75), with a trend toward increased odds of SGA (OR 2.37, 95% CI 0.97-5.79). P-interactions were suggestive of an interaction by infant sex. Sensitivity models additionally adjusting for gestational age at delivery did not substantively change the results.

**Table 4:** Associations between prenatal negative life events (NLEs) and adverse birth outcomes stratified by sex: Logistic regression^a^

NLEs	All Infants		Girls^b^		Boys^b^		p-interaction
	OR	95% CI	OR	95% CI	OR	95% CI	
Outcome: Low birth weight (LBW)^c^							
0-2	Ref	NA	Ref	NA	Ref	NA	0.07
> 2	2.11	0.89-5.02	**4.44**	**1.11-17.76**	0.85	0.25-2.92	
Outcome: Small for gestational age (SGA)^d^							
0-2	Ref	NA	Ref	NA	Ref	NA	0.03
> 2	1.42	0.73-2.76	2.37	0.97-5.79	0.61	0.20-1.81	
Outcome: Any adverse birth outcome^e^							
0-2	Ref	NA	Ref	NA	Ref	NA	0.04
> 2	1.48	0.80-2.78	**2.41**	**1.01-5.75**	0.81	0.31-2.15	

^a^ Multivariable model adjusted for [infant sex,] cluster, ethnicity, maternal marriage status, age, weight and height; and family home ownership

^b^ LBW with NLE > 2: Girls *N* = 24, Boys *N* = 13; SGA with NLE > 2: Girls *N* = 38, Boys *N* = 16; Any adverse birth outcome with NLE > 2: Girls *N* = 41, Boys *N* = 22

^c^ LBW defined as birth weight < 2500 g

^d^ SGA defined as birth weight < 10th percentile for gestational age

^e^ Any adverse birth outcome defined as an LBW, SGA, or stillborn infant

Text in bold have odds ratios significantly different from 1

## Discussion

Our prospective study adds to the literature supporting a relationship between prenatal maternal stress and both infant birth anthropometrics and adverse pregnancy outcomes, suggesting that these relationships operate not only in higher-income countries but also in rural lower- and middle-income countries (LMICs), including Ghana. Specifically, our data demonstrate an exposure-response relationship between increased prenatal maternal stress and reduced birth length in all infants and an exposure-response relationship between increased prenatal maternal stress and reduced birth weight and head circumference in girls. Further, girls exposed to high levels of prenatal maternal stress were at increased likelihood of having an adverse birth outcome.

Impairments in birth anthropometrics predict infant mortality, which are particularly prevalent in LMICs. While genetics certainly play a role, the importance of non-chemical prenatal environmental exposures is increasingly apparent [25]. Our study enrolled non-smoking women and these data suggest a reduction in birth length of 0.9 cm in infants born to women reporting high prenatal stress, nearly three times the reduction reported with second hand tobacco smoke exposure [26]. In girls, an average reduction in birth weight of 170 g and 2-fold increased odds of LBW, SGA or stillborn infant was seen, similar to reductions found with active maternal smoking [27, 28]. Notably, cigarette smoking is associated with a 150% increase in overall perinatal mortality. Understanding the role of non-chemical exposures, such as maternal stress, on fetal development will help identify at-risk infants and influence development of preventative efforts.

In the study population as a whole, our data suggest that increased prenatal maternal stress is associated with reduced birth length. The placenta, the maternal-fetal interface, buffers the impact of maternal environmental exposures to promote fetal development. Maternal stress increases maternal cortisol levels and induces inflammation, impacting placental activity [29]. The placental response to maternal cortisol involves 11 *β*-hydroxysteroid dehydrogenase (11 *β*-HSD) enzymes and placental glucocorticoid receptor (GR) expression, and may be overwhelmed resulting in increased fetal cortisol exposure. Sheep exposed to a late gestation increase in cortisol have a significant reduction in biometric measurements of size [30]. Animal models show that mice prenatally exposed to maternally-administered betamethasone have reductions in birth anthropometrics [31, 32]. Increased fetal cortisol exposure may also alter programming the fetus’ developing HPA axis – a finding demonstrated in both animal and human studies – with future health implications [33–36].

Our data suggest that girls are particularly vulnerable to the effects of prenatal maternal stress. The placenta is sexually dimorphic and may influence the impact of maternal stress differently in male versus female fetuses [37]. For example, female as compared to male placentas respond to maternal stress with decreased 11 *β*-HSD2 [33], increased 11 *β*-HSD1 [38], and altered GR expression [39] resulting in decreased placental cortisol metabolism and increased fetal cortisol exposure in females [40]. In females, prenatally administered glucocorticoids altered 11 *β*-HSD2 activity and were associated with increased cord blood cortisol [41]. A study of maternal asthma in pregnancy demonstrated that maternal inflammation reduced placental 11 *β*-HSD2 activity and increased cord blood cortisol in female fetuses, resulting in reduced fetal growth [33]. Further, significant reductions in 11 *β*-HSD2 and increases in 11 *β*-HSD1 activity have been demonstrated in intrauterine growth-restricted female placentas [42].

The finding that prenatal maternal stress is associated with impaired birth anthropometrics may have health effects beyond the perinatal period and may influence infant and adult health. Support for this hypothesis, known as DOHaD, originated from observation that fetal growth restriction correlated with subsequent high adult mortality, including cardiovascular, pulmonary, oncologic and cerebrovascular mortality [43]. However, these risks are not absolute. For example, postnatal influences such as diet have been shown to modify the association between birth weight and cardiovascular disease [44]. Therefore, understanding risk factors associated with impaired birth anthropometrics could help identify at-risk infants and guide implementation of future preventative efforts.

Strengths of this study include the prospective study design, non-smoking pregnant women and prenatally assessed maternal stress across multiple domains. Further, despite the rural LMIC nature of the cohort, we confirmed gestational age at enrollment via ultrasound and measured birth anthropometrics within 24 h of birth regardless of location of delivery (i.e., home versus clinic). These data are the first in rural sub-Saharan Africa to elucidate the relationship between prenatal maternal stress and birth outcomes and build upon previous reports from higher-income countries linking maternal stress and birth outcomes. If replicated, given the importance of these birth outcomes in predicting infant mortality, our findings suggest new targets for public health intervention. Other strengths include the focus on a rural, LMIC population more likely to be impacted by perinatal mortality and impaired birth anthropometrics and our well-characterized cohort that allowed us to adjust for a number of important confounders and pathway variables.

We also acknowledge limitations. Chronic maternal stress over the prior 6 months was assessed across 11 domains, but it is possible that additional stressors were present and not evaluated. Temporal associations to identify sensitive windows of exposure are unable to be explored, as we did not serially measure maternal stress across gestation. While we adjust for many important confounders, we did not have data on paternal height or co-varying environmental exposures such as maternal diet. Our study does not include biomarkers of maternal stress or placental analyses therefore we can only hypothesize potential mechanistic pathways. Future studies would be strengthened by the incorporation of biomarkers of both maternal and fetal stress response systems, including HPA axis function. It also would be useful to have additional longitudinal follow up, to determine the childhood health ramifications of prenatal maternal stress in this population.

## Conclusions

In summary, these data demonstrate that all infants exposed to prenatal maternal stress are at increased risk for decreased birth length and that girls are particularly vulnerable to the effects of maternal stress on birth weight and head circumference, with increased risk of adverse birth outcomes. Given the direct links between birth anthropometrics and perinatal mortality, specifically in LMICs, and morbidity and mortality across the life course, these data begin to elucidate at-risk groups and highlight potential future public health interventions. Counseling and validation of the importance of managing maternal stress during pregnancy may be a simple intervention that can raise awareness to the perinatal health effects of maternal stress.

## Supplementary information

**Additional file 1: Table S1.** Reported negative life events (NLEs) domains of CRYSIS-R survey. **Table S2.** Associations between prenatal negative life events (NLEs) and adverse birth outcomes stratified by sex: Sensitivity model additionally adjusting for gestational age

## Data Availability

The dataset used during the current study is available from the corresponding author on reasonable request.

## Abbreviations

CRISYS-R: Crisis in Family Systems-Revised
GRAPHS: Ghana Randomized Air Pollution and Health Study
KHRC: Kintampo Health Research Centre
LBW: Low birth weight
LMICs: Lower- and middle-income countries
NLEs: Negative life events
SGA: Small-for-gestational age
