# Supplementary material for: Prenatal maternal stress and birth outcomes in rural Ghana: sex-specific associations
Source: BMC Pregnancy Childbirth. 2019 Oct 29;19:391. doi: 10.1186/s12884-019-2535-9 (PMC6819589; doi:10.1186/s12884-019-2535-9)
Supplement: Supplementary file 1 — Additional file 1: Table S1. Reported negative life events (NLEs) domains of CRYSIS-R survey. Table S2. Associations between prenatal negative life events (NLEs) and adverse birth outcomes stratified by sex: Sensitivity model additionally adjusting for gestational age [file 12884_2019_2535_MOESM1_ESM.docx]

| **Table S1.** Reported negative life events (NLEs) domains of CRYSIS-R survey | |
| --- | --- |
| Domain | N (%) |
| Financial | 286 (81.02) |
| Legal | 34 (9.63) |
| Career | 25 (7.08) |
| Relationship | 236 (66.86) |
| Home safety | 92 (26.06) |
| Neighbourhood safety | 131 (37.11) |
| Medical, Self | 75 (21.25) |
| Medical, Others | 151 (42.78) |
| Home issues | 247 (69.97) |
| Prejudice | 75 (21.25) |
| Difficulties with authorities | 7 (1.98) |
| Other | 220 (62.32) |

| **Table S2.** Associations between prenatal negative life events (NLEs) and adverse birth outcomes stratified by sex: Sensitivity model additionally adjusting for gestational age | | | | | | | | | |
| --- | --- | --- | --- | --- | --- | --- | --- | --- | --- |
| NLEs | All Infants^a^ | |  | Girls^b^ | |  | Boys^b^ | | p-interaction |
|  | OR | 95% CI |  | OR | 95% CI |  | OR | 95% CI |  |
| **Outcome: LBW^c^** | | | | | | | | | |
| 0-2 | Ref | NA |  | Ref | NA |  | Ref | NA | 0.07 |
| >2 | 2.23 | 0.92-5.43 |  | **4.30** | **1.07-17.30** |  | 1.41 | 0.32-6.27 |  |
| **Outcome: SGA^d^** | | | | | | | | | |
| 0-2 | Ref | NA |  | Ref | NA |  | Ref | NA | 0.02 |
| >2 | 1.40 | 0.73-2.74 |  | 2.34 | 0.96-5.73 |  | 0.60 | 0.20-1.80 |  |
| **Outcome: Any adverse birth outcome^e^** | | | | | | | | | |
| 0-2 | Ref | NA |  | Ref | NA |  | Ref | NA | 0.05 |
| >2 | 1.52 | 0.81-2.85 |  | 2.42 | 1.01-5.80 |  | 0.91 | 0.34-2.47 |  |
| ^a^ Multivariable model adjusted for infant sex, gestational age at delivery**,** ethnicity, cluster, maternal marriage status, age, weight and height; and family home ownership  ^b^ LBW with NLE>2: Girls N=24, Boys N=13; SGA with NLE>2: Girls N=38, Boys N=16; Any adverse birth outcome with NLE>2: Girls N=41, Boys N=22  ^c^ LBW = birth weight < 2500 grams  ^d^ SGA= birth weight <10^th^ percentile for gestational age  ^e^ Any adverse birth outcome, defined as any LBW, SGA, or stillborn infant | | | | | | | | | |
